# Anti-Inflammation of Spirocyclopiperazinium Salt Compound LXM-10 Targeting α7 nAChR and M4 mAChR and Inhibiting JAK2/STAT3 Pathway in Rats

**DOI:** 10.1371/journal.pone.0066895

**Published:** 2013-06-28

**Authors:** Weiwei Zhang, Qi Sun, Xiaoli Gao, Yimin Jiang, Runtao Li, Jia Ye

**Affiliations:** 1 State Key Laboratory of Natural and Biomimetic Drugs, Peking University, Beijing, China; 2 Department of Molecular and Cellular Pharmacology, School of Pharmaceutical Sciences, Peking University, Beijing, China; 3 Department of Chemical Biology, School of Pharmaceutical Sciences, Peking University, Beijing, China; 4 Medical and Healthy Analysis Center, Peking University, Beijing, China; University of North Dakota, United States of America

## Abstract

The present study aims to investigate the therapeutic effects of LXM-10 by intragastric administration in both acute and chronic inflammatory models, and to explore the underlying molecular mechanisms. The results showed that LXM-10 produced significant anti-inflammatory effects on carrageenan induced paw edema and complete Freund's adjuvant (CFA) induced arthritis, in which LXM-10 inhibited paw swelling in a dose- and time-dependent manner. ELISA analysis showed the production of pro-inflammatory cytokines including TNF-α and IL-6 was decreased by LXM-10. Western blot analysis showed that LXM-10 significantly reduced phosphorylation of Janus kinase 2 (JAK2) and further blunted phosphorylation of signal transducer and activator of transcription-3 (STAT3). The effects that LXM-10 had shown were attenuated by methyllycaconitine citrate (an α7 nicotinic acetylcholine receptor antagonist) or tropicamide (an M4 muscarinic acetylcholine receptor antagonist) in vivo. In conclusion, the studies showed that intragastric administration of LXM-10 exerted significant anti-inflammation effects in acute and chronic models, which may be attribute to the activation of α7 nicotinic acetylcholine receptor and M4 muscarinic acetylcholine receptor, thereby inhibiting the JAK2/STAT3 signal pathway, and ultimately reducing the production of pro-inflammatory cytokines of TNF-α and IL-6.

## Introduction

Inflammation is an essential immune response in warding off invading pathogens and injury, but sometimes it becomes one of major causes of human morbidity and mortality, which results from the excessive or persistent production and release of cytokines in orchestrating an infection or injury. The uncontrolled inflammatory response has been implicated in the pathogenesis of multiple diseases such as endotoxemia, rheumatoid arthritis, Crohn's disease, type 2 diabetes and atherosclerosis [1], [2]. Traditional treatments for controlling the inflammation are the steroidal and non-steroidal anti-inflammatory drugs, but many side effects limit their utility [3], [4]. Recently, cholinergic anti-inflammatory pathway provides a major advantage to the design of novel pharmacological strategies against inflammatory diseases, in which acetylcholine is released from vagus nerve terminals and activates the α7 nicotinic acetylcholine receptor expressed on macrophages and other cytokine-producing cells, controlling pro-inflammatory cytokine synthesis and preventing tissue damage.

**Figure 1:**
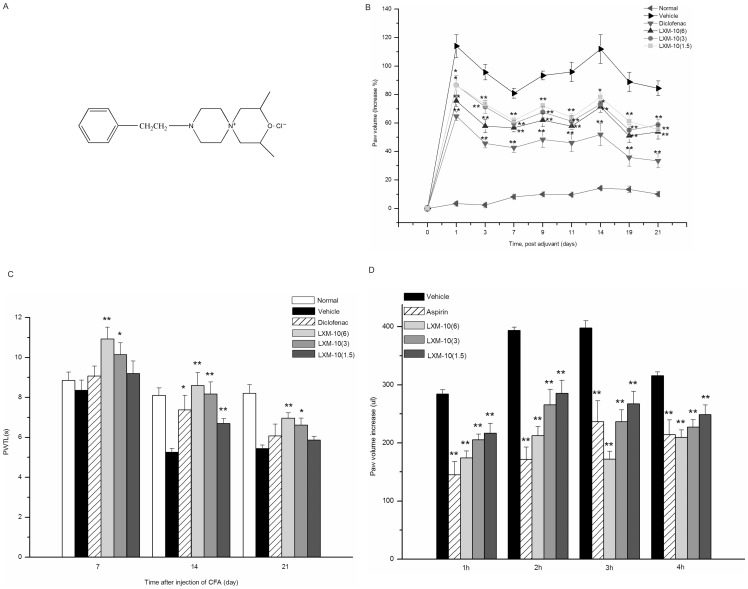
The chemical structure of LXM-10 and its anti-inflammatory effects in vivo. **(A) The chemical structure of the spirocyclopiperazinium compound LXM-10. (B) Effect of LXM-10 on complete Freund's adjuvant (CFA)-induced paw swelling.** The rats were administered with LXM-10 (6, 3, 1.5 mg/kg, i.g.), diclofenac (5 mg/kg, i.g.) or vehicle for 21 days after injecting CFA. The paw volume was evaluated on days 0, 1, 3, 7, 9, 11, 14, 19 and 21. All data are mean ± SEM of 6 rats per group. Significant differences from the vehicle group at the same time are indicated by ^*^ *P*<0.05 and ^**^ *P*<0.01. (**C**) **Effect of LXM-10 on complete Freund's adjuvant (CFA)-induced thermal hyperalgesia.** The rats were administered with LXM-10 (6, 3, 1.5 mg/kg, i.g.), diclofenac (5 mg/kg, i.g.) or vehicle for 21 days after injecting CFA. The thermal hyperalgesia were detected on days 7, 14, 21. All data are mean ± SEM of 6 rats per group. Significant differences from the vehicle group at the same time are indicated by ^*^ *P*<0.05 and ^**^ *P*<0.01. (**D**) **Effect of LXM-10 on carrageenan induced paw oedema.** The rats were administered with LXM-10 (6, 3, 1.5 mg/kg, i.g.), aspirin (300 mg/kg, i.g.), or vehicle (i.g.) and the paw volume was measured at 1, 2, 3 and 4 h after challenged by carrageenan. All data are mean ± SEM of 8 rats per group. Significant differences from the vehicle group at the same time are indicated by ^*^ *P*<0.05 and ^**^ *P*<0.01.

The compound LXM-10 (2, 4-dimethyl-9-β-phenylethyl-3-oxo-6, 9-diazaspiro [5.5]undecane chloride), a spirocyclopiperazinium salt compound (Fig. 1A), showed no obvious classical nicotinic or muscarinic responses in mice, and the median lethal dose (LD50) was 510 mg/kg [5]. Previous studies showed that pretreatment of LXM-10 prevented acute inflammation, which may be related to α7 nicotinic acetylcholine receptor (α7 nAChR) and M4 muscarinic acetylcholine receptor (M4 mAChR) [6]. However, its effect on chronic inflammation and the molecular mechanism that what signal pathway is invovled in after activating α7 nAChR or M4 mAChR remain unclear. The present study is to evaluate the activity of LXM-10 by intragastric administration in both acute and chronic inflammation, and further explore the possible mechanisms and the underlying anti-inflammatory signaling pathway.

## Materials and Methods

### Animals

Healthy adult male Sprague-Dawley rats (weighing 180∼200 g) were used throughout the experiment and were provided by the Department of Laboratory Animal Science of Peking University. This animal study was approved by the Institutional Animal Care and Use Committee of Peking University, and all animal procedures were performed according to the IACUC policy. All efforts were made to minimize animals suffering, and to reduce the number of animals used. All rats were housed under standardized animal house conditions (on a 12 h light/dark cycle at 23±1°C, and relative humidity 55±5%) and with free access to commercial rat diet and water.

### Drugs and reagents

Drug uses: Methyllycaconitine citrate (MLA), tropicamide, aspirin, carrageenan and complete Freund's adjuvant (CFA) (Sigma Chemical Co., St. Louis, MO, USA), Diclofenac Sodium (Beijing Novartis Pharma Ltd, PR China). LXM-10 was synthesized by Runtao Li and Qi Sun. All drugs were dissolved in distilled water immediately before use in a volume of 10 ml/kg by intragastric injection (i.g.), intraperitoneal injection (i.p.) or intradermal injection (i.d.).

Reagents and antibodies: RIPA lysis buffer (Beijing Beyotime Institute of Biotechnology, PR China), protease inhibitor cocktails, phosphatase inhibitors, BCA Protein assay kit (Beijing Applygen Technologies Inc. PR China), polyvinylidene fluoride membrane (Millipore, Bedford, MA, USA), anti-STAT3 antibody, anti-phospho-STAT3 (Tyr705) antibody (Cell Signaling Technology, Danvers, MA, USA), anti-JAK-2 antibody, anti-JAK2 (phospho Y1007+ Y1008) antibody (Abcam, Cambridge, MA, USA), anti-β-actin antibody, anti-mouse IgG and anti-rabbit IgG horseradish peroxidase-linked (HRP) antibodies (Santa Cruz, CA, USA), Rat IL-6 Quantikine ELISA Kit, Rat TNF-alpha Quantikine ELISA Kit (R&D Systems, Inc., Minneapolis, MN, USA).

### Adjuvant-induced arthritis

The rats were randomly divided into four groups: normal group, vehicle group, LXM-10 (6, 3, 1.5 mg/kg/day, i.g.), and diclofenac sodium (5 mg/kg/day, i.g.). Arthritis was evoked by an intradermal injection of 0.1 ml complete Freund's adjuvant (CFA) in the foot pad of right hind limb of rats [7]. Paw volume was measured before as well as on days 1, 3, 7, 9, 14, 19 and 21 after CFA injection using a digital plethysmometer (model YLS-78, Shandong Academy of Medical Sciences, PR China). Percentages of swelling compared with the initial paw volumes were tracked and the inhibition ratios were calculated. The inflammatory thermal hyperalgesia was evaluated by hot plate test on the days 7, 14 and 21, the hot plate (Model GJ-8401, P.R. China) maintained at 52±0.2°C and the paw withdrawal thermal latency (PWTL) was determined as the duration from start of the thermal stimulation to the occurrence of the hindpaw withdrawal reflex and the cut-off time was set at 30 s. The pain threshold elevated rate (PTE) was used as a measurement index and calculated by the following formula.

### Carrageenan-induced paw edema

Rats were injected subcutaneously in the right hind paw with 100 μl of 1% carrageenan solution. LXM-10 (6, 3, 1.5 mg/kg, i.g.), aspirin (300 mg/kg, i.g.), or vehicle was given immediately after carrageenan injection. The paw volume was measured with a plethysmometer (model YLS-78, Shandong Academy of Medical Sciences, PR China) before and 1, 2, 3 and 4 h after carrageenan induction. The percentage inhibition of edema was calculated by the following formula.

Inhibition (%)  =  [(edema volume of vehicle group − edema volume of experimental group)/edema volume of vehicle group] ×100.

To elucidate the anti-inflammatory mechanisms of LXM-10, methyllycaconitine (MLA, an α7 nicotinic acetylcholine receptor antagonist, 3 mg/kg, i.p.), tropicamide (Tro, an M4 muscarinic acetylcholine receptor antagonist, 3 mg/kg, i.p.) or vehicle was injected 20 min prior to LXM-10 (6 mg/kg, i.g.), respectively.

### Tissue preparation

As previously described, the animals were sacrificed at 3 h after carrageenan injection, paws were cut at the level of the calcaneus bone, and paw tissues were weighed and frozen at −80°C until used.

### Western blot analysis

Paw tissues were rapidly homogenized in ice-cold RIPA lysis buffer (1∶7.5, w/v) containing 1% protease inhibitors and 1% phosphatase inhibitors, then centrifugated at 10 000×g for 30 min. The supernatant was collected and stored at −80°C as total protein for western analysis. Protein concentration was determined by BCA protein assay kit according to the manufacturer's instructions.

Protein aliquots (50 µg) were separated by 10% SDS-PAGE, and electrotransferred onto a polyvinylidene fluoride membrane, and then blocked with 5% BSA for 1.5 h at room temperature. Antibodies for JAK-2, P-JAK-2 (Y1007+ Y1008), STAT3, P-STAT3 (Tyr705) and β-actin were diluted in Tris-Buffered-Saline with Tween-20 (TBST) (1∶1000∼1∶5000) and rocked overnight at 4°C, followed by incubation with specific horseradish peroxidase (HRP)-conjugate secondary antibody. Blots were developed with Biorad ChemiDoc XRS (BIO-RAD, USA) and the optical density analysis of signals was quantified using Quantity One software (Version 4.5, Bio-Rad Laboratories, Inc., Hercules, CA, USA).

### Cytokines Assay

The tissues were homogenized in PBS (1∶2, w/v) containing 1% protease inhibitors and then centrifuged at 12 000×g for 15 min at 4°C. The supernatants were analyzed for TNF-α and IL-6 using ELISA kit according to the manufacturer's instructions.

### Statistical analysis

All the data were expressed as mean ± SEM. One-way ANOVA was performed for the statistical analysis of data, the Student's t test was used to analyze statistical significance and *P*<0.05 was considered to be statistically significant.

## Results

### Anti-inflammatory effects of LXM-10 in rats

#### On CFA-induced arthritis

Fig. 1B illustrated that after injection of CFA, there was a gradual increase of swelling in the injected paw of the rats compared with the normal group (*P*<0.01), reaching a maximal swelling of 114% and the average was 98% within 21 days. LXM-10 dose-dependently inhibited the edema formation compared with the vehicle group (*P*<0.01, *P*<0.05); the maximum inhibition ratios were 43%, 38% and 31% at the doses of 6, 3 and 1.5 mg/kg respectively on day 19. Diclofenac reduced paw volume by 60% at the same time (*P*<0.01).

There was a pronounced thermal hyperalgesia in the rats after injection of CFA (*P*<0.01, *P*<0.05) (Fig. 1C). Treated with LXM-10 (6, 3, 1.5 mg/kg, i.g.), the paw withdrawal thermal latency (PWTL) increased significantly in experimental period within 21 days (*P*<0.01, *P*<0.05), and the pain threshold elevated rates (PTEs) were 64%, 56% and 28% on day 14. Diclofenic (5 mg/kg, i.g.) increased the PWTL by 41% at the same time.

#### On carrageenan-induced edema

As shown in Fig. 1D, injection of carrageenan induced a rapid and marked paw edema in rats which was peaked at 3 h and lasted 4 h. LXM-10 showed significant reduction of rat paw edema compared with the vehicle group (*P*<0.01), in a dose- and time-dependent manner. After administration of LXM-10 (6, 3, 1.5 mg/kg, i.g.), the paw edema was reduced by 57%, 41% and 33% respectively at 3 h, while aspirin (300 mg/kg, i.g.) was 41% at the same time and 56% at 2 h.

### Mechanisms of anti-inflammation of LXM-10 in vivo

#### Involvement of α7 nicotinic acetylcholine receptor and M4 muscarinic acetylcholine receptor

**Figure 2:**
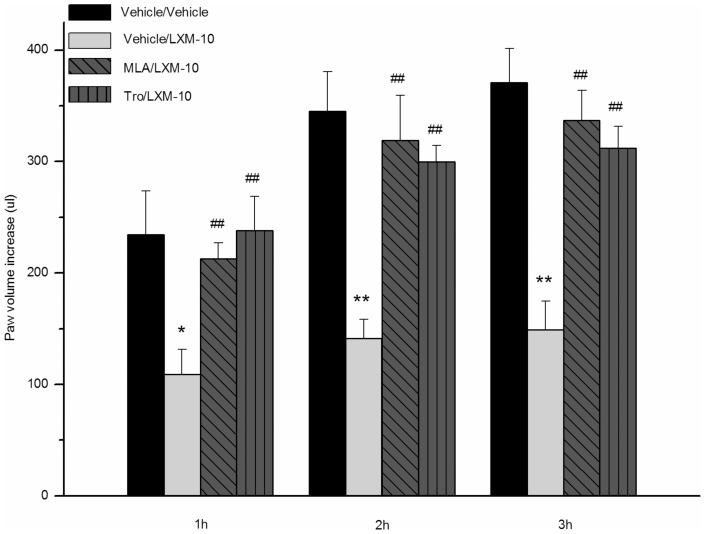
Effect of LXM-10 on carrageenan induced rat paw edema was attenuated by methyllycaconitine citrate and tropicamide. The rats were treated with methyllycaconitine citrate (MLA, 3 mg/kg, i.p.), tropicamide (Tro, 3 mg/kg, i.p.) or vehicle before administration of LXM-10 (6 mg/kg, i.g.) or vehicle. The paw volume was measured at 1, 2 and 3 h after challenged by carrageenan. All data are mean ± SEM of 8 rats per group. ^*^ *P*<0.05 and ^**^ *P*<0.01 vs Vehicle/Vehicle; ^##^ *P*<0.01 vs Vehicle/LXM-10.

As shown in Fig. 2, when pretreated with methyllycaconitine citrate (3 mg/kg, i.p.) or tropicamide (3 mg/kg, i.p.), the anti-inflammatory effect of LXM-10 was attenuated in carrageenan-induced paw edema test. The inhibition ratios were reduced to 9%, 8% or 9% by methyllycaconitine citrate, and to −2%, 13% or 16% by tropicamide at 1 h, 2 h or 3 h.

#### On TNF-α and IL-6 formation

**Figure 3:**
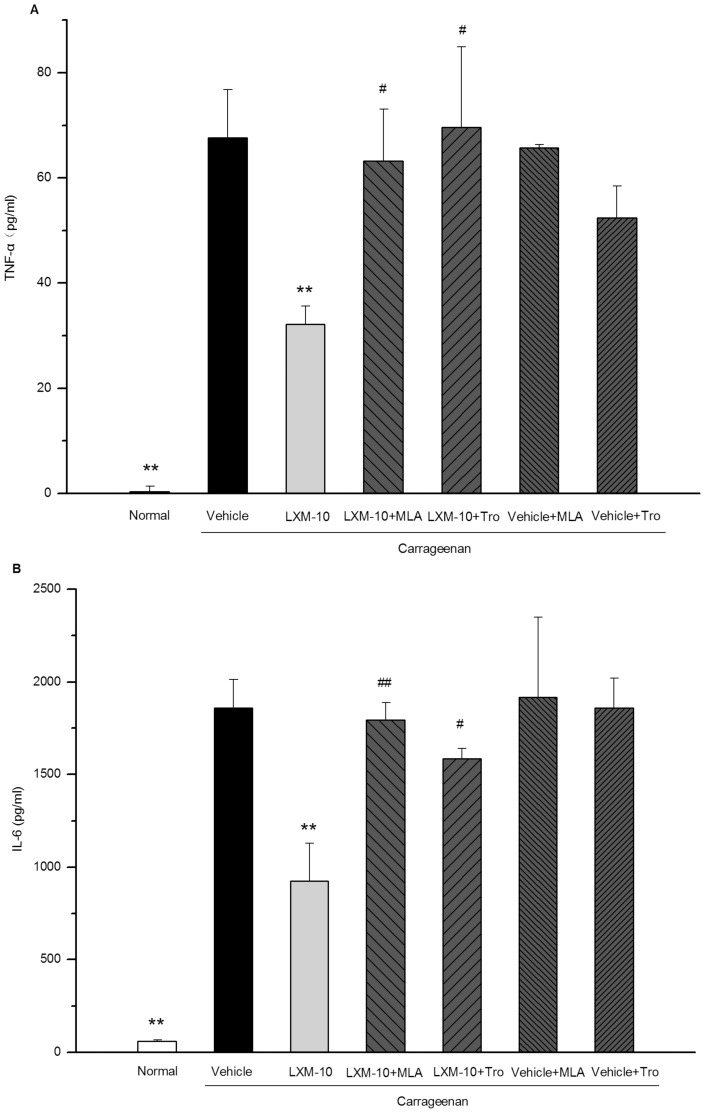
LXM-10 induced suppression of TNF-α and IL-6 was attenuated by methyllycaconitine citrate and tropicamide. Rats were treated with methyllycaconitine citrate (MLA, 3 mg/kg, i.p.), tropicamide (Tro, 3 mg/kg, i.p.) or vehicle prior to administration of LXM-10 (6 mg/kg, i.g.) or vehicle, and the levels of TNF-α (**A**) and IL-6 (**B**) were measured in hind paws at 3 h after carrageenan challenge. All data are mean ± SEM of 6∼8 rats per group. ^**^ *P*<0.01 vs Vehicle and ^#^ *P*<0.05 vs LXM-10 at the same time point.

As shown in Fig. 3A, carrageenan induced a sharp rise of TNF-α in the injected paws from 0.28 to 68 pg/ml (*P*<0.01), LXM-10 (6 mg/kg, i.g.) markedly decreased the level of TNF-α (*P*<0.01), with the inhibition ratio of 52%. Methyllycaconitine citrate or tropicamide attenuated the effect to 7% or −3% respectively (*P*<0.05).

Consistent with the effect on the TNF-α assay, LXM-10 (6 mg/kg, i.g.) also reduced the production of IL-6 by 50% (*P*<0.01). Methyllycaconitine citrate or tropicamide attenuated the effect to 3% or 15%, respectively (P<0.05). (Fig. 3B).

#### On the expression of P-JAK2

**Figure 4:**
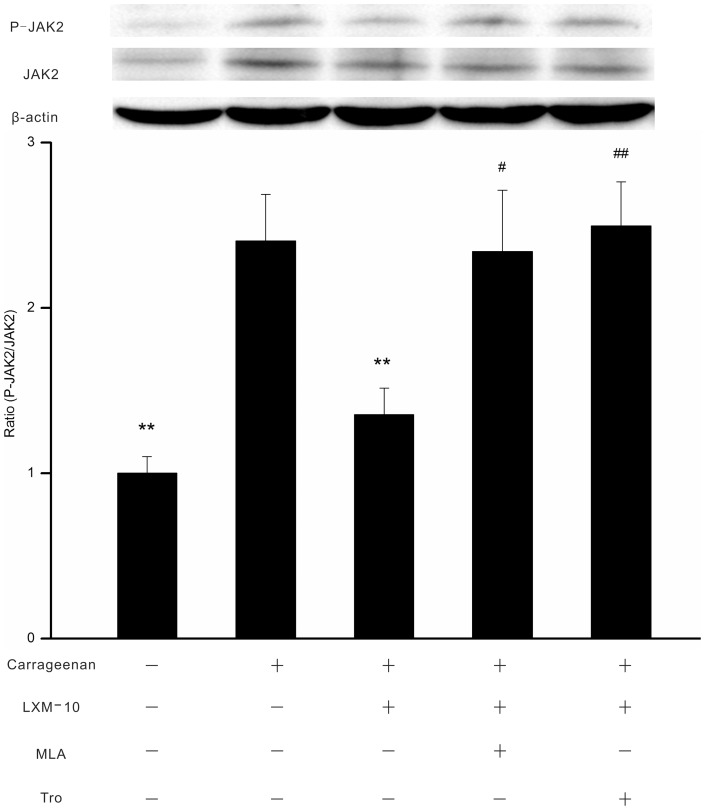
Effect of LXM-10 on expression of P-JAK2 was attenuated by methyllycaconitine citrate and tropicamide. The rats were treated with methyllycaconitine citrate (MLA, 3 mg/kg, i.p.), tropicamide (Tro, 3 mg/kg, i.p.) or vehicle before administration of LXM-10 (6 mg/kg, i.g.) or vehicle. The expression of JAK2 and P-JAK2 in hind paws was detected at 3 h after carrageenan challenge. All data are mean ± SEM of 6 rats per group. ^**^ *P*<0.01 vs Vehicle; ^#^ *P*<0.05 and ^##^ *P*<0.01 vs LXM-10.

As shown in Fig. 4, carrageenan induced a pronounced increase in the phosphorylation of JAK2 in paw tissues compared with the normal group (*P*<0.01). LXM-10 (6 mg/kg, i.g.) inhibited the expression of P-JAK2 (*P*<0.01), and the effect was attenuated by methyllycaconitine citrate (3 mg/kg, i.p.) or tropicamide (3 mg/kg, i.p.) (*P*<0.01, *P*<0.05).

#### On the expression of P-STAT3

**Figure 5:**
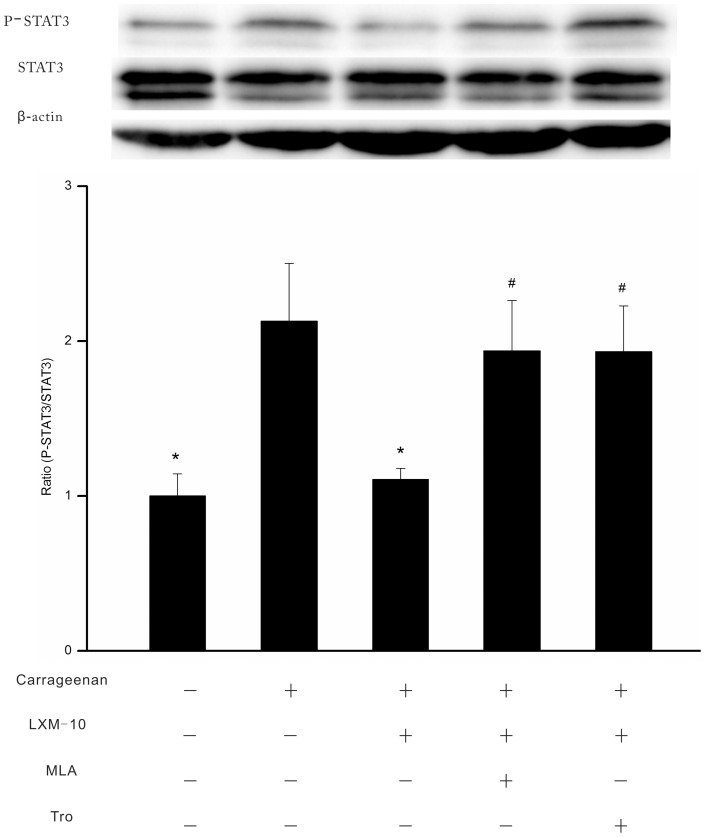
Effect of LXM-10 on expression of P-STAT3 was attenuated by methyllycaconitine citrate and tropicamide. The rats were treated with methyllycaconitine citrate (MLA, 3 mg/kg, i.p.), tropicamide (Tro, 3 mg/kg, i.p.) or vehicle before administration of LXM-10 (6 mg/kg, i.g.) or vehicle. The expression of STAT3 and P-STAT3 in hind paws was detected at 3 h after carrageenan challenge. All data are mean ± SEM of 6 rats per group. ^*^ *P*<0.05 vs Vehicle, ^#^ *P*<0.05 vs LXM-10.

There was a marked up-regulation of the expression of phosphorylated STAT3 in paw tissues when challenged by carrageenan (Fig. 5) (*P*<0.05). LXM-10 (6 mg/kg, i.g.) inhibited the expression of P-STAT3 significantly (*P*<0.05), and the effect was attenuated by methyllycaconitine citrate (3 mg/kg, i.p.) or tropicamide (3 mg/kg, i.p.) (*P*<0.05).

## Discussion

Inflammation is a complex set of interactions among soluble factors and cells that can arise in any tissue in response to traumatic, infectious, post-ischaemic, toxic or autoimmune injury [8]. When acute inflammation escalates out of control, it may cause serious sepsis, which is the leading cause of death [9]. Moreover, the acute inflammation may lead to persistent damage and switch to chronic inflammation, which is often associated with many pathophysiologic processes and diseases, such as cancer, atherosclerosis, osteoarthritis and Alzheimer's disease [10], [11]. Inflammation and inflammation-mediated illnesses are the biggest challenges in current medicine, understanding and resolving inflammation is therefore currently one of the main targets in medical science.

Recently, a novel neural pathway termed as cholinergic anti-inflammatory reflex has been discovered, which inhibits the production of inflammatory cytokines. Many cholinergic agonists have been designed to develop as novel anti-inflammatory drugs and some of them have shown anti-inflammatory effects in vivo and in vitro, such as GTS-21 and CAP55 [12]. However, their usability is limited for their lack of specificity. Spirocyclopiperazinium salt compound LXM-10 is a cholinergic agonist; previous studies showed that pretreatment of LXM-10 (s.c.) exerted anti-inflammatory effect in acute models with no obvious side-effects [6], [13]. In the present study, we evaluated the therapeutic effects of LXM-10 in both acute and chronic inflammatory models by intragastric administration, and further explored the molecular mechanisms. Complete Freund's adjuvant (CFA) induced arthritis has been used as a model of sub-chronic or chronic inflammation, which showed considerable correlation with rheumatoid arthritis in human [14], [15]. CFA elicits joint swelling, synovial membrane inflammation, cartilage destruction, and increases the sensitivity of pressure or thermal stimulus in the paw. The study showed that intragastric administration of LXM-10 inhibited paw swelling and inflammatory thermal hyperagesia in CFA induced arthritis, and reduced rat paw edema in carrageenan-evoked inflammation, indicating LXM-10 exerted suppressive effects on acute and chronic inflammation.

α7 nicotinic acetylcholine receptor (α7 nAChR) is one subtype of nicotinic acetylcholine receptors and plays a vital role in mediating cholinergic anti-inflammatory pathway. α7 nAChR is expressed on different types of cells such as neurons, macrophages, lymphocytes, monocytes, neutrophils, dendritic cells, etc. [16]. Activation of the α7 nAChR expressed on resident macrophages may suppress the local inflammation by reducing the production of pro-inflammatory cytokines TNF and IL-6 [17], [18], which are closely associated with some inflammatory diseases, including sepsis, rheumatoid arthritis, asthma, and diabetes [19]–[21]. It appears that the α7 nAChR is a promising target for developing novel anti-inflammatory drugs. Though there is no clinically approved drug primarily targeted on α7 nAChR, some novel α7 nAChR agonists have been reported to exhibit significant anti-inflammatory effect and seem to have potential for the clinical trails. CNI-1493, served as a selective pro-inflammatory cytokine inhibitor [22], has shown effect to resolve endotoxic shock in a rat model [23]; GTS-21 is able to attenuate pro-inflammatory cytokines production and improve survival rate of mice with endotoxemia [24]. M4 muscarinic acetylcholine receptor (M4 mAChR) is classified as the same group with M2 muscarinic receptor for coupling to Gi/o. M4 mAChR is predominantly expressed in the central nervous system [25], and some inflammatory cells like neutrophils, eosinophils and macrophagocytes [20], [26], [27]. Previous study showed that the anti-inflammation of LXM-10 was related to α7 nAChR and M4 mAChR in Xylene-induced ear edema in mice [6]. To further confirm whether the α7 nAChR and M4 mAChR are involved in anti-inflammatory mechanisms of LXM-10, we used methyllycaconitine citrate (an α7 nicotinic acetylcholine receptor antagonist), and tropicamide (an M4 muscarinic acetylcholine receptor antagonist) to investigate the anti-inflammation of LXM-10 in carrageenan induced paw edema in rats. The results showed that the effect was attenuated by methyllycaconitine citrate or tropicamide, indicating the anti-inflammation mechanisms of LXM-10 invovled the activation of α7 nAChR and M4 mAChR.

α7 nAChR agonists control systemic inflammation in sepsis by inhibiting the JAK2/STAT3 pathway which plays an important role in transducing a multitude of inflammatory signals; it has been considered as a therapeutic target for suppressing inflammatory process [28]–[30]. The JAK2 is an essential tyrosine kinase for modulating the immune response, which contributes to “early” inflammatory response in sepsis when phosphorylated at Tyr1007/1008 [31]–[35]. The phosphorylated JAK2 activates transcription factor STAT3 by phosphorylation at Tyr705; phosphorylated STAT3 entering the nucleus, resulting in a transcriptional response, which is critical for the production of pro-inflammatory cytokines, such as IL-6 and IL-1β [36]. Inhibition of JAK2 and STAT3 tyrosine phosphorylation may reduce TNF-α level in sepsis in vivo [28], [29], indicating TNF-α is also involved in the JAK2/STAT3 pathway in anti-inflammation. Reports showed cholinergic agonists inhibited the production of pro-inflammation cytokines via activating the α7 nAChR expressed on macrophages and/or splenocytes [20], [21], [37], and resulting in the inhibition of the JAK2-induced STAT3 tyrosine phosphorylation [28], [29]. However, there is no evidence showing that activation of M4 muscarinic receptor contributes to blunting the JAK2/STAT3 signal pathway and producing anti-inflammation.

To investigate whether the JAK2/STAT3 signal pathway is involved in the anti-inflammation of LXM-10, we tested the expression of P-JAK2 and P-STAT3 (Tyr705) in the edema paws induced by carrageenan. The results showed that the tyrosine phosphorylation of JAK2 and STAT3 were inhibited by LXM-10, and the effects were blocked by methyllycaconitine citrate or tropicamide, which suggested that LXM-10 probably suppressed the inflammation via acting α7 nAChR or M4 mAChR, and sequentially inhibiting the phosphorylation of JAK2 and reducing the signal transduction of STAT3. The result indicated that the anti-inflammation of LXM-10 was related to inhibition of the JAK2/STAT3 pathway.

One of the leading causes of unrestrained inflammation is the excessive production of pro-inflammatory cytokines, such as TNF-α and IL-6. TNF-α, a primary and potent mediator of inflammation, plays a critical role in the inflammatory response and is capable of causing end-organ dysfunction that occurs in severe sepsis. IL-6 is one of the most important mediators of fever and of the acute-phase response [38], and plays a key role in several inflammatory diseases including rheumatoid arthritis, systemic juvenile idiopathic arthritis, systemic lupus erythematosus, ankylosing spondylitis, etc. [39]. Our results showed that LXM-10 significantly reduced the production of TNF-α and IL-6, and the effects were attenuated by methyllycaconitine citrate or tropicamide, which demonstrated that the anti-inflammation of LXM-10 was probably mediated by suppressing the production of TNF-α and IL-6.

In summary, we found that intragastric administration of LXM-10 exerted significant anti-inflammation in acute and chronic inflammation models, and the mechanism was mediated by activation of α7 nicotinic acetylcholine receptor or M4 muscarinic receptor, inhibiting the JAK2/STAT3 signaling pathway, and reducing the production of pro-inflammatory cytokines TNF-α and IL-6. In the present studies, we first found that activating M4 receptor may suppress the JAK2/STAT3 signaling pathway and exert anti-inflammation effect. Meanwhile, we first reported the activation of JAK2/STAT3 pathway was involved in inflammation model of carrageenan induced paw edema. For future studies, we will focus on exploring the relationship between the α7 nAChR and the M4 mAChR in the process of anti-inflammation.
